# Analysis of Molecular Interactions between Components in Phospholipid-Immunosuppressant-Antioxidant Mixed Langmuir Films

**DOI:** 10.1021/acs.langmuir.1c00434

**Published:** 2021-04-29

**Authors:** Małgorzata Jurak, Klaudia Szafran, Pilar Cea, Santiago Martín

**Affiliations:** † Department of Interfacial Phenomena, Institute of Chemical Sciences, Faculty of Chemistry, Maria Curie-Skłodowska University, 20031 Lublin, Poland; ‡ Instituto de Nanociencia y Materiales de Aragón (INMA), CSIC-Universidad de Zaragoza, 50009 Zaragoza, Spain; § Departamento de Química Física, Facultad de Ciencias, Universidad de Zaragoza, 50009 Zaragoza, Spain

## Abstract

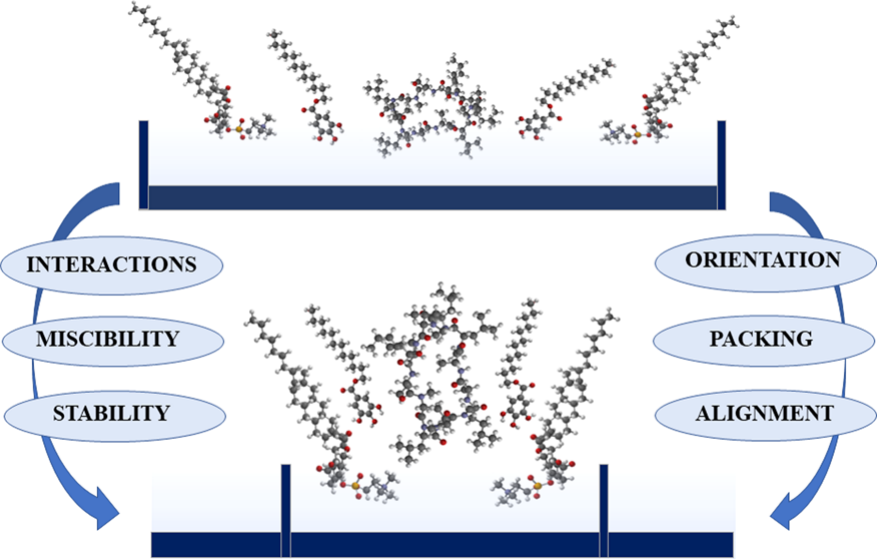

The study of Langmuir monolayers incorporating biomimetic and bioactive substances plays an important role today in assessing the properties and quality of the molecular films for potential biomedical applications. Here, miscibility of binary and ternary monolayers of phospholipid (dioleoyl phosphatidylcholine, DOPC), immunosuppressant (cyclosporine A, CsA), and antioxidant (lauryl gallate, LG) of varying molar fractions was analyzed by means of the Langmuir technique coupled with a surface potential (Δ*V*) module at the air–water interface. The surface pressure–area per molecule (π–*A*) isotherms provided information on the physical state of the films at a given surface pressure, the monolayer packing and ordering, and the type and strength of intermolecular interactions. Surface potential–area (Δ*V*–*A*) isotherms revealed the molecular orientation changes at the interface upon compression. In addition, the apparent dipole moment of the monolayer-forming molecules was determined from the surface potential isotherms. The obtained results indicated that the film compression provoked subsequent changes of CsA conformation and/or orientation, conferring better affinity for the hydrocarbon environment. The mutual interactions between the components were analyzed here in terms of the excess and total Gibbs energy of mixing, whose values depended on the stoichiometry of the mixed films. The strongest attraction, thus the highest thermodynamic stability, was found for a DOPC–CsA–LG mixture with a 1:1:2 molar ratio. Based on these results, a molecular model for the organization of the molecules within the Langmuir film was proposed. Through this model, we elucidated the significant role of LG in improving the miscibility of CsA in the model DOPC membrane and thus in increasing the stability of self-assembled monolayers by noncovalent interactions, such as H-bonds and Lifshitz–van der Waals forces. The above 1:1:2 combination of three components is revealed as the most promising film composition for the modification of implant device surfaces to improve their biocompatibility. Further insight into mechanisms concerning drug–membrane interactions at the molecular level is provided, which results in great importance for biocoating design and development as well as for drug release at target sites.

## Introduction

**Scheme 1:**
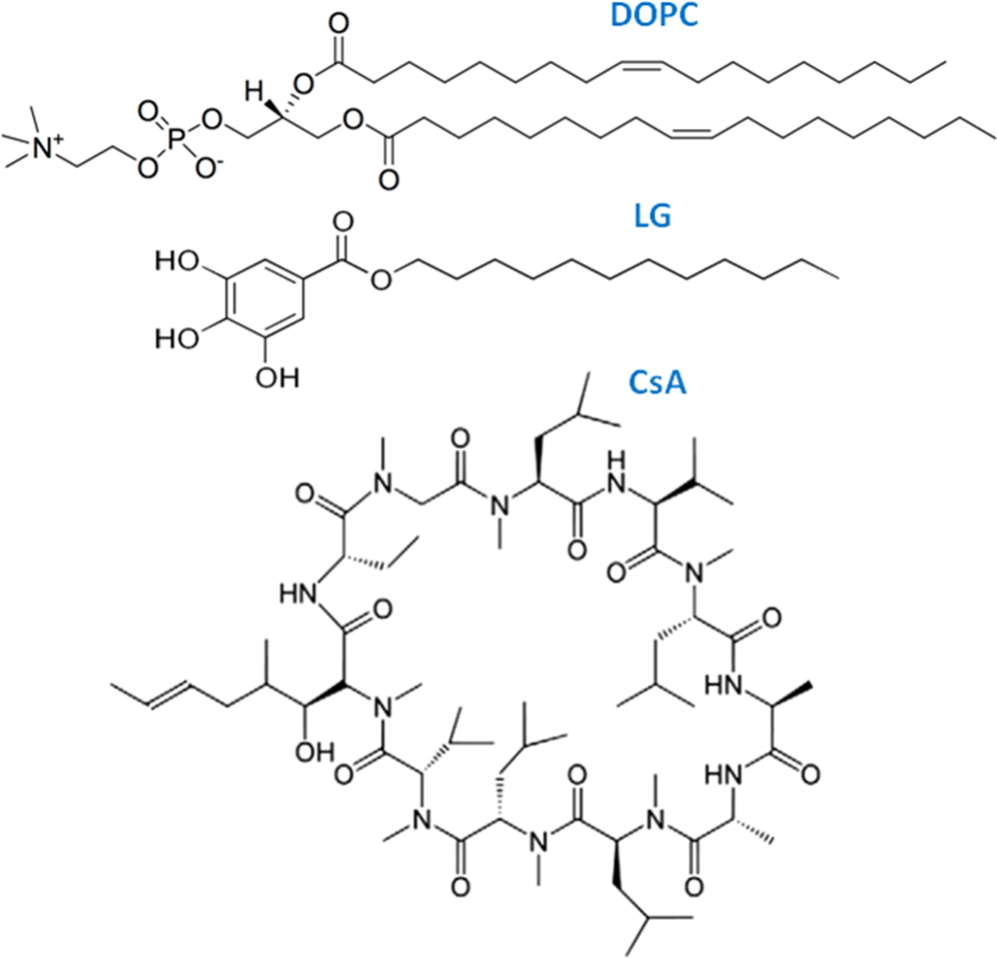
Chemical Structures of 1,2-Dioleoyl-*sn*-glycero-3-phosphocholine (DOPC), Lauryl Gallate (LG), and Cyclosporine A (CsA)

Ultrathin films characterized by high homogeneity, continuity, defined composition, and chemical structure as well as defined stability and wettability are used to modify the surface properties of implants. In this context, the physicochemical characteristics of Langmuir (L) and Langmuir-Blodgett (LB) monolayers, well-known ultrathin films, are part of intensive research in biomimetic systems.^1−3^ The integration of these ultrathin films with the tissue strictly depends on the immune response of the organism, which is determined, among others, by the degree of biocompatibility of the material with cells.^4^ One of the ways to improve biocompatibility is to modify the implant surface with a biocompatible living tissue layer of the desired physicochemical properties, which would prevent activation of the immune system, infection, and rejection of the implant.^2,3^ In this aspect, there is a need for the preparation and characterization of multicomponent Langmuir films containing cell-friendly components of natural biological membranes (phospholipids, PL), as well as compounds with immunosuppressive activity (cyclosporine A, CsA) and antioxidant (lauryl gallate, LG). Their chemical structures are presented in Scheme 1.

Phospholipids with the choline moiety (phosphatidylcholines, PCs) are the most abundant class in eukaryotic cells.^5^ PCs bearing a *cis*-9 double bond, such as 1,2-dioleoyl-*sn*-glycero-3-phosphocholine (DOPC), represent a major fraction in all biomembranes. The presence of compounds that build natural biological membranes is a specific link between the artificial and biological systems, increasing the probability of a positive response of the organism, and at the same time, it can facilitate the introduction and then the release of an active substance such as CsA.

CsA is a cyclic polypeptide used to suppress immune responses due to its selective lymphocyte inhibition action. CsA is employed to block intracellular signal-transduction in T-lymphocytes to prevent organ transplant from rejection as well as in the treatment of several autoimmune disorders.^6^ Beyond immunosuppressive function, CsA also exhibits a variety of biological activities, including antifungal, anti-inflammatory, and antiparasitic properties.^6,7^ The unique amino acid (4*R*)-4-[(*E*)-butenyl]-4,*N*-dimethyl-l-threonine (MeBmt) is known to be involved in CsA biological activity.^8^

Despite the promising applications of CsA, this material is a neutral, extremely hydrophobic drug of high molecular weight (1203 Da), which exhibits low water solubility, poor permeability through biological barriers (gastrointestinal tract, skin, and cornea), and instability in the gastrointestinal medium. Thus, the administration of this sparingly water-soluble drug is either complicated or ineffective through the oral route.^9,10^ Therefore, other ways of introducing CsA into the body are being sought. The direct coating of the implant surface with the CsA film is a route that deserves exploration. Moreover, the major clinical concern is CsA-induced nephrotoxicity, hepatotoxicity, neurotoxicity, and cardiovascular diseases.^6,10−12^ These side-effects are associated with reactive oxygen species (ROS). Namely, CsA action/treatment generates excessive production of oxygen free radicals that lead to lipid peroxidation, which is the main source of damage to the cell membrane integrity, e.g., in vascular and cardiac tissues,^13^ general inflamed tissues, and implanted biomaterials. Previous studies have shown that scavenging of ROS by a well-known antioxidant, gallic acid, inhibits lipid peroxidation and protects the heart and lysosome membrane against oxidative stress.^14^ Furthermore, the combination of gallic acid with CsA more effectively affects cardiac performance and the reduction of infarct size.^15^ The protective role of different antioxidants is also confirmed in CsA-induced hepatotoxicity^16,17^ and nephrotoxicity.^18,19^ Accordingly, the incorporation of an antioxidant in association with CsA can be an effective way to reduce the undesirable effects of CsA, protect unsaturated PL bonds against oxidation, and consequently reduce the risk of implant rejection. A potential candidate can be one of the derivatives of gallic acid, i.e., lauryl gallate (LG), which in contrast to the former compound is capable of forming the water-insoluble Langmuir monolayers.

It is well-known that LG exhibits both potent chain breaking and preventive antioxidant activity by capturing free radicals.^20,21^ It prevents the generation of superoxide radicals by xanthine oxidase, inhibiting the enzyme. Beyond the antioxidizing activity, LG has also been reported to possess antibacterial activity against Gram-positive microorganisms by inhibiting the respiratory chain in bacteria and anticarcinogenic effects on animal models or human cell lines.^21−25^ These properties can be largely associated with its amphiphilic structure. The LG molecules have a polar pyrogallol (PG) moiety (three −OH groups with an aromatic ring) connected by an ester bond with a hydrophobic alkyl chain (C12). LG is capable of capturing free radicals by donating the phenolic hydrogen atom in the aromatic rings,^26^ while hydrophobic alkyl chain facilitates to reside in such sites of the lipid core of membranes where it is needed. The amphiphilic structure and low water solubility favor the organization of LG molecules at the interfaces. From the wide spectrum of gallates, only lauryl gallate (LG) optimizes high antioxidant activity with sufficient hydrophobicity^27^ to form a stable and compressible monolayer at the air–water interface (Langmuir film). Nevertheless, the properties of these types of modifying layers, being the combination of the phospholipid DOPC, cyclosporine A, and lauryl gallate, have not been described in the literature so far.

Here, we report a comprehensive physicochemical characterization of binary and ternary Langmuir films containing DOPC, CsA, and LG of different molar fractions. Excess area, excess Gibbs energy, and total Gibbs energy of mixing provide information about the stoichiometry of the mixture with the highest thermodynamic stability. In addition, the surface potential–area (Δ*V*–*A*) isotherms are indicative of changes in the orientation of the molecules, revealing that a change of apparent dipole moments at the interface is highly dependent on the mixed film composition. Expanding knowledge on this topic could help in the development of a more rational and scientific approach to the design of biocompatible coatings containing biologically active compounds.

## Materials and Methods

### Materials

1,2-Dioleoyl-*sn*-glycero-3-phosphocholine (DOPC, ≥99%, Sigma), cyclosporine A (CsA, ≥99%, Alfa Aesar), and lauryl gallate (LG, ≥99%, Aldrich) were used as received. The appropriate amounts of the above compounds were dissolved in a chloroform/methanol (4:1, v/v) mixture to obtain a final concentration of 1 mg/mL. Chloroform was purchased from Macron Fine Chemicals (99.8%) and methanol from Fluka (≥99.9%). Then, the binary (DOPC–LG, CsA–LG, DOPC–CsA) and ternary (DOPC–LG–CsA, CsA–LG–DOPC, DOPC–CsA–LG) systems were prepared by mixing proper volumes of basic solutions so as to receive the molar fractions of the second or third component, respectively, equal to 0.25, 0.50, and 0.75. In addition, for the ternary mixtures, the constant molar ratio of two components 1:1 was maintained.

### Methods

The surface pressure–area per molecule (π–*A*) and surface potential–area per molecule (Δ*V*–*A*) isotherms were registered on a pure water subphase (Millipore Milli-Q purification system, resistivity 18.2 MΩ cm) using a Nima Teflon trough (720 × 100 mm^2^) contained in a constant temperature (20 ± 1 °C) clean room. The surface pressure was measured using the Wilhelmy paper plate with an accuracy of 0.1 mN/m. The solutions (35–65 μL) were spread using a microsyringe (Hamilton–Bonaduz, Switzerland) on the water subphase, and the solvent was allowed to evaporate over 10 minutes before starting the compression of the film with the trough barriers moving at a rate of 29 cm^2^/min. Each isotherm was repeated at least three times to confirm its reproducibility. Simultaneously, the surface potential–area per molecule isotherms were registered using a Kelvin Probe provided by Nanofilm Technologie GmbH, Göttingen, Germany.

## Results and Discussion

The Langmuir technique is a unique method for preparing monomolecular insoluble films of biological substances on aqueous phases, in which intermolecular interactions as well as their influence on the molecular alignment can be easily determined.

The unsaturated phospholipid DOPC (Scheme 1) was used as a membrane model. Using such a less complicated system is very useful to gain information on the binding of proteins to membranes highly dependent on the mutual interactions. Several studies have shown that the poorly water-soluble CsA penetrates lipid membranes showing affinity to the gel/fluid boundaries and disrupts the order of acyl chains, especially around the polar heads.^28,29^ Its interactions with phospholipids (DPPC, POPC, DPPE, DPPS, DPPG)^3,30−33^ with a dominant repulsive character generate no miscibility or only partial miscibility. Therefore, the incorporation of the LG antioxidant, which interacts with both DOPC and CsA, can increase the miscibility of these two components and improve the mixture stability.

In the first stage, and after evaluating single monolayers, binary systems with well-defined molar fractions (*x* = 0.25, 0.50, 0.75) of CsA, DOPC, and LG will be studied. Subsequently, the ternary systems in which the molar ratio of two components is kept constant (1:1) while varying the molar ratio of the third component will be discussed.

### Surface Pressure–Area (π–*A*) Isotherms

#### Single Monolayers

**Figure 1:**
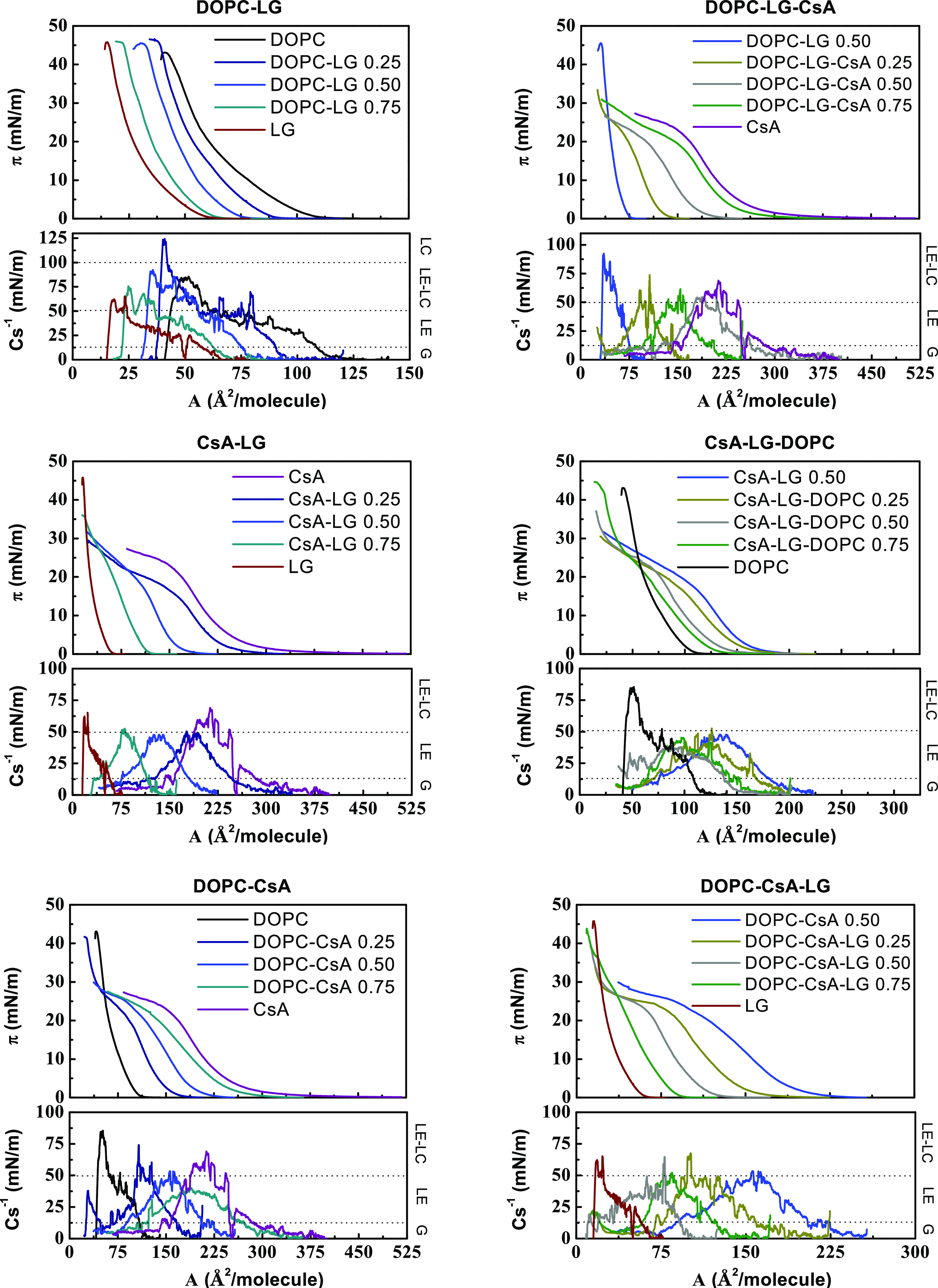
Surface pressure–area per molecule (π–*A*) isotherms and compression modulus-area per molecule (*C*_s_^–1^–*A*) graphs for the single, binary, and ternary monolayers for the indicated molar fractions of components.

Each of the compounds studied here (DOPC, CsA, and LG) forms true Langmuir monolayers at the air–water interface, as shown in Figure 1, with the isotherms being consistent with the data published previously.^3,34^ Here, the “true” term means that the layer-forming molecules are practically insoluble in the water subphase and these molecules are capable of forming a two-dimensional film at the interface upon the compression process. The π–*A* curves exhibit a small slope, which is characteristic of high monolayer compressibility. From these isotherms, the take-off area, *A*_0_, has been determined. *A*_0_ denotes the first value for the area per molecule at which the surface pressure can be detected, i.e., π ≅ 0.5 mN/m, upon the compression process, and it corresponds to the transition from a gas to an expanded liquid phase. The “isotherm take-off” takes place at different area per molecule values, *A*_0_, depending on the compound (*A*_0,CsA_ = 380.4 Å^2^, *A*_0,DOPC_ = 115.2 Å^2^, and *A*_0,LG_ = 60.3 Å^2^); remarkably *A*_0,CsA_ is 3 and 6 times greater than *A*_0,DOPC_ and *A*_0,LG_, respectively. These isotherms indicate that the monolayers possess a two-dimensional liquid-like organization, i.e., a liquid-expanded (LE) phase. In the most packaged state, the limit area, *A*_lim_, estimated by extrapolating the linear part of the isotherm to the zero surface pressure, for the monolayers is as follows: *A*_lim,CsA_ = 260.9 Å^2^, *A*_lim,DOPC_ = 77.8 Å^2^, and *A*_lim,LG_ = 35.3 Å^2^. The *A*_lim,CsA_ value is in good agreement with a single CsA molecule with the dimensions of 16.1 × 12.4 Å^2^ along the longer axis.^30^ Since *A*_lim,CsA_ (260.9 Å^2^) is greater than the cross-sectional area for the CsA ring arranged perpendicular to the surface plane, i.e., 182 Å^2^,^35^ and lower than the area per molecule in a flat orientation, i.e., 374 Å^2^ (assuming the area of CsA as a circle), it is likely that CsA molecules lay with their rings rather inclined toward the surface. Therefore, taking into account the indicated area per molecule values, an average tilt angle of the CsA molecule with respect to the surface plane could be estimated at ca. 50°. Meanwhile, the *A*_lim,DOPC_ and *A*_lim,LG_ values are consistent with a vertical orientation with respect to the air–water interface for the DOPC and LG molecules.

Finally, the collapse surface pressure value was determined by projection on the *y*-axis (π) of the intersection point of the two lines being extensions of the isotherm below and above its inflection (collapse). The CsA monolayer collapses at a surface pressure ca. twice lower (π_c,CsA_ = 23.1 mN/m) than the surface pressure of a monolayer of DOPC or LG (π_c,DOPC_ = 43.3 mN/m and π_c,LG_ = 45.9 mN/m). These data are gathered in Table 1.

**Table 1:** Take-Off Area (*A*_0_), Limit Area (*A*_lim_), and Collapse Surface Pressure (π_c_) for Single, Binary, and Ternary Monolayers^a^

binary monolayers					ternary monolayers				
	*x*	*A*_0_ (Å^2^)	*A*_lim_ (Å^2^)	π_c_ (mN/m)		*x*	*A*_0_ (Å^2^)	*A*_lim_ (Å^2^)	π_c_ (mN/m)
DOPC–LG	0	115.2	77.8	43.3	DOPC–LG–CsA	0	75.8	56.6	45.4
	0.25	89.5	63.5	45.9		0.25	138.6	122.0	21.5
	0.50	75.8	56.6	45.4		0.50	211.3	176.4	19.1
	0.75	64.1	44.9	45.6		0.75	296.6	237.8	18.9
	1	60.3	35.3	45.9		1	380.4	260.9	23.1
CsA–LG	0	380.4	260.9	23.1	CsA–LG–DOPC	0	192.8	165.3	19.4
	0.25	281.7	232.5	18.5		0.25	178.0	151.9	18.6
	0.50	192.8	165.3	19.4		0.50	154.5	126.2	22.6
	0.75	119.1	105.5	33.8		0.75	136.3	118.2	22.3/42.5
	1	60.3	35.3	45.9		1	115.2	77.8	43.3
DOPC–CsA	0	115.2	77.8	43.3	DOPC–CsA–LG	0	223.7	197.0	22.8
	0.25	173.8	154.0	22.7/41.9		0.25	176.5	146.3	23.0
	0.50	223.7	197.0	22.8		0.50	127.9	110.5	23.0
	0.75	299.1	247.9	22.5		0.75	91.8	77.8	43.6
	1	380.4	260.9	23.1		1	60.3	35.3	45.9

a *x* denotes the molar fraction of the last (second or third) component in the mixtures.

#### Binary and Ternary Monolayers

The isotherms registered for the DOPC–LG, CsA–LG, and DOPC–CsA mixed monolayers lie between those obtained for individual components, with *A*_0_ and *A*_lim_ showing intermediate values (Figure 1 and Table 1). This dependence is similar for the isotherms of ternary monolayers (DOPC–LG–CsA, CsA–LG–DOPC, DOPC–CsA–LG), although here the effect of adding different amounts of the third component (CsA, DOPC, LG, respectively) is analyzed with respect to the binary monolayer with a constant equimolar ratio of the other two components (Figure 1 and Table 1). The π – *A* isotherms of all monolayers show the liquid-expanded phase characteristics in the whole range of surface pressure, with the *plateau* typical of the expanded liquid-condensed liquid 1st order phase transition not being observed in any of the isotherms. This observation is further confirmed by the compressibility modulus data, as explained below.

### Compressibility Modulus

To analyze the effect of composition on molecular packing in the mixed monolayers in more detail, the compression modulus values, *C*_s_^–1^ = *f*(π), were calculated directly from the π–*A* isotherm data using eq 1(36)
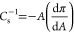
1The obtained values (Figures 1 and S1) provide information about the physical state of monolayers strictly associated with the packing and ordering of molecules at the air–water interface. According to the Davies and Rideal classification, the liquid-expanded (LE) state is characterized by the Young’s modulus values between 12.5 and 50 mN/m, while the liquid-condensed (LC) state by those between 100 and 250 mN/m.^36^ In addition, the *C*_s_^–1^ values within the limits of 0–12.5 and 50–100 mN/m can be indicative of the gas (G) phase and the LE-LC transition, respectively. These regimes are presented in Figure 1. For the sake of clarity, the data at selected surface pressures are summarized in Table 2.

**Table 2:** Compression Modulus *C*_s_^–1^ Determined at 5, 10, and 15 mN/m and its Maximum Value with the Corresponding Surface Pressure, *C*_s,max_^–1^/π

		*C*_s_^–1^ for binary monolayers (mN/m)						*C*_s_^–1^ for ternary monolayers (mN/m)			
	*x*	5	10	15	*C*_s,max_^–1^/π		*x*	5	10	15	*C*_s,max_^–1^/π
DOPC–LG	0	33	48	49	85/30	DOPC–LG–CsA	0	39	56	69	92/38
	0.25	50	61	47	124/40		0.25	44	46	42	74/6
	0.50	39	56	69	92/38		0.50	35	43	38	62/7
	0.75	31	41	43	76/40		0.75	26	48	46	55/12
	1	24	30	34	65/26		1	47	66	58	69/11
CsA–LG	0	47	66	58	69/11	CsA–LG–DOPC	0	37	47	41	48/11
	0.25	34	44	33	50/12		0.25	32	39	36	53/8
	0.50	37	47	41	48/11		0.50	30	34	41	46/13
	0.75	36	47	51	53/13		0.75	29	36	36	38/10
	1	24	30	34	65/26		1	33	48	49	85/30
DOPC–CsA	0	33	48	49	85/30	DOPC–CsA–LG	0	37	53	36	53/10
	0.25	33	47	63	74/15		0.25	33	41	48	68/16
	0.50	37	53	36	53/10		0.50	32	45	50	52/12
	0.75	33	38	33	40/10		0.75	36	41	34	65/4
	1	47	66	58	69/11		1	24	30	34	65/26

The maximum values of *C*_s_^–1^ correspond to the most compressed state of the monolayer that is manifested as the “peak” point of the *C*_s_^–1^ = *f*(*A*) function (Figure 1). Based on these maximum *C*_s_^–1^ values, it can be claimed that all monolayers are in a liquid-expanded (LE) state in line with the Davies and Rideal criterion.^36^ Only for higher molar fractions of DOPC in the systems, a more condensed phase is achieved (Table 2).

In the surface pressure range of 5–15 mN/m, among single monolayers, the CsA film is characterized by the highest values of *C*_s_^–1^ within 47–66 mN/m. Meanwhile, for the DOPC monolayer, the values are lower, between 33 and 49 mN/m, and for LG between 24 and 34 mN/m. Hence, it can be concluded that CsA forms the less flexible films, in agreement with the lowest collapse surface pressure obtained for this monolayer. This observation may be explained in terms of intramolecular hydrogen bonds due to the presence of four available amide groups, which contribute to the rigidity of the cyclic skeleton.^6,9^ On the other hand, the LG monolayer is the most loosely packed. It is worth mentioning that LG is a polyphenol comprising three hydroxyl groups capable of forming an extensive intermolecular hydrogen bond network; in addition, LG contains aromatic rings, which may result in intermolecular π–π stacking.^37^ Such noncovalent interactions can provoke the long-range orientational order in the head group region, where presumably one aryl ring can make edge-to-face arrangements with four neighboring aryl rings.^38^ Nevertheless, the relatively large polar pyrogallol groups impede the hydrocarbon tails to be packed closely in the monolayer and the chains can be tilted to optimize their Lifshitz-van der Waals interactions, contributing to the monolayer fluidity. In turn, in DOPC unsaturated bonds in the *cis* conformation promote chain disorder and formation of the structure with an intermediate fluidity between the CsA and LG monolayers.^34^

As expected, since all monolayers of the single components are in a LE phase, the mixed films also retain the same physical phase albeit showing very peculiar packing density changes and an irregular pattern (Table 2). In the DOPC–LG, CsA–LG, and DOPC–CsA monolayers, the values of compression modulus range from 31 to 69 mN/m. The highest value was obtained for the mixed film DOPC–LG 0.50 at 15 mN/m, with a *C*_s_^–1^ value of 69 mN/m. In addition, the compression modulus of the ternary mixtures reaches lower values compared to those of the binary mixtures in the 26–50 mN/m range. Hence, the general tendency is that the increase in the number of components hinders the creation of more packed films although at a well-defined molar ratio, the film can be stiffer due to specific alignment of molecules and their interactions; for instance, the DOPC–CsA–LG 0.50 monolayer at 15 mN/m exhibits the highest value of *C*_s_^–1^ among the ternary systems, with *C*_s_^–1^ = 50 mN/m. The increased *C*_s_^–1^ obtained for the above-mentioned binary and ternary mixtures indicate the increased packing of molecules in relation to the other monolayers, which is indicative of stronger attractive interactions between the molecules. This observation is further confirmed by the analysis of the excess Gibbs energy, which reaches more negative values at the given component ratios as it will be shown later.

### Collapse Surface Pressure versus Composition (π_c_–*x*)

As mentioned above, the DOPC and LG monolayers collapse at higher surface pressures than the CsA monolayer (Figure 1 and Table 1). Valuable information about the miscibility of components in a mixed film can be obtained from the collapse surface pressure of the monolayers. Here, DOPC–LG mixed monolayers exhibit a collapse surface pressure close to the LG monolayer (Figure 1), which reveals the stability of the mixtures even at high surface pressures, as reported previously.^34^ However, for the CsA-containing monolayers (DOPC–CsA 0.25, CsA–LG–DOPC 0.75) with a low CsA molar fraction, two kinks are visible (Figure 1). The lower corresponds to the collapse surface pressure of CsA, and the higher one to that of DOPC (Table 1). However, at higher CsA molar fractions, the second collapse cannot be observed for all mixed monolayers due to technical limitations. The trough surface is limited and with a low DOPC or LG content in the monolayer, the full isotherm cannot be recorded, even when the barriers reach the completely closed position. Similar observations were reported before for mixtures of CsA with other lipids.^33^ Independent collapses in isotherms of mixed monolayers, which appear at the same surface pressure as the breakdown of monolayers of the pure components, are indicative of immiscibility between the components and their strong tendency toward phase segregation, with the component having a lower collapse surface pressure being expelled from the monolayer. When the components are partially miscible, they may form domains integrated mainly either by one or the other component. Once the surface pressure of collapse of CsA is reached, the domains rich in CsA also collapse. The other possibility is that the components can mix and interact below the first collapse pressure, and only above this pressure they become immiscible and CsA is expelled from the monolayer. To gain insight into the behavior of CsA with DOPC and/or LG at surface pressures below the first collapse, further analysis is conducted based on thermodynamic functions (mean molecular areas in the mixed binary (*A*_12_) or ternary monolayers (*A*_123_), excess area (*A*_exc_), excess Gibbs energy changes (Δ*G*_exc_), and total Gibbs energy of mixing changes (Δ*G*_mix_) parameters of interaction have been calculated). Their negative deviations from ideality can be considered as a criterion of the monolayer stability, while positive deviations can point out the phase separation in the monolayer.^39,40^

### Miscibility

Miscibility and interactions between molecules can be analyzed in accordance with the additivity rule.^39,40^ Namely, mean molecular areas in the mixed binary (*A*_12_) or ternary monolayers (*A*_123_) at given surface pressures were designated directly from the π–*A* isotherms and collated to those gained for the ideal miscibility or complete immiscibility of molecules (eq 2)
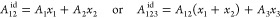
2where *A*_1_, *A*_2_, and *A*_3_ denote the mean molecular areas at a given surface pressure in the one-component films and *x*_1_, *x*_2_, and *x*_3_ are the molar fractions of ingredients 1, 2, and 3 in the mixed films, respectively.

Then, to indicate the type of possible interactions (attraction or repulsion) between molecules in the binary and ternary monolayers, the excess area per molecule (*A*_exc_) was determined using eq 3
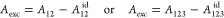
3Excess areas equal to zero are indicative of either miscible or totally immiscible films, while negative excess areas are indicative of stronger attraction forces between the components in the monolayer than those acting between molecules in the pure compounds, although negative excess areas may also be due to steric effects (e.g., insertion of one molecule in the structure of the other one to form a complex). In addition, the magnitude of these interactions can be evaluated through the excess Gibbs energy of mixing, Δ*G*_exc_ (eq 4)
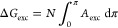
4with *N* being the Avogadro’s number.

Finally, the thermodynamic stability of the mixed systems was characterized based on the total Gibbs energy of mixing, Δ*G*_mix_ (eq 5)
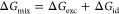
5where the ideal Gibbs energy of mixing, Δ*G*_id_, can be expressed as
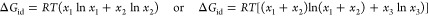
6with *R* being the gas constant and *T* the temperature.

**Figure 2:**
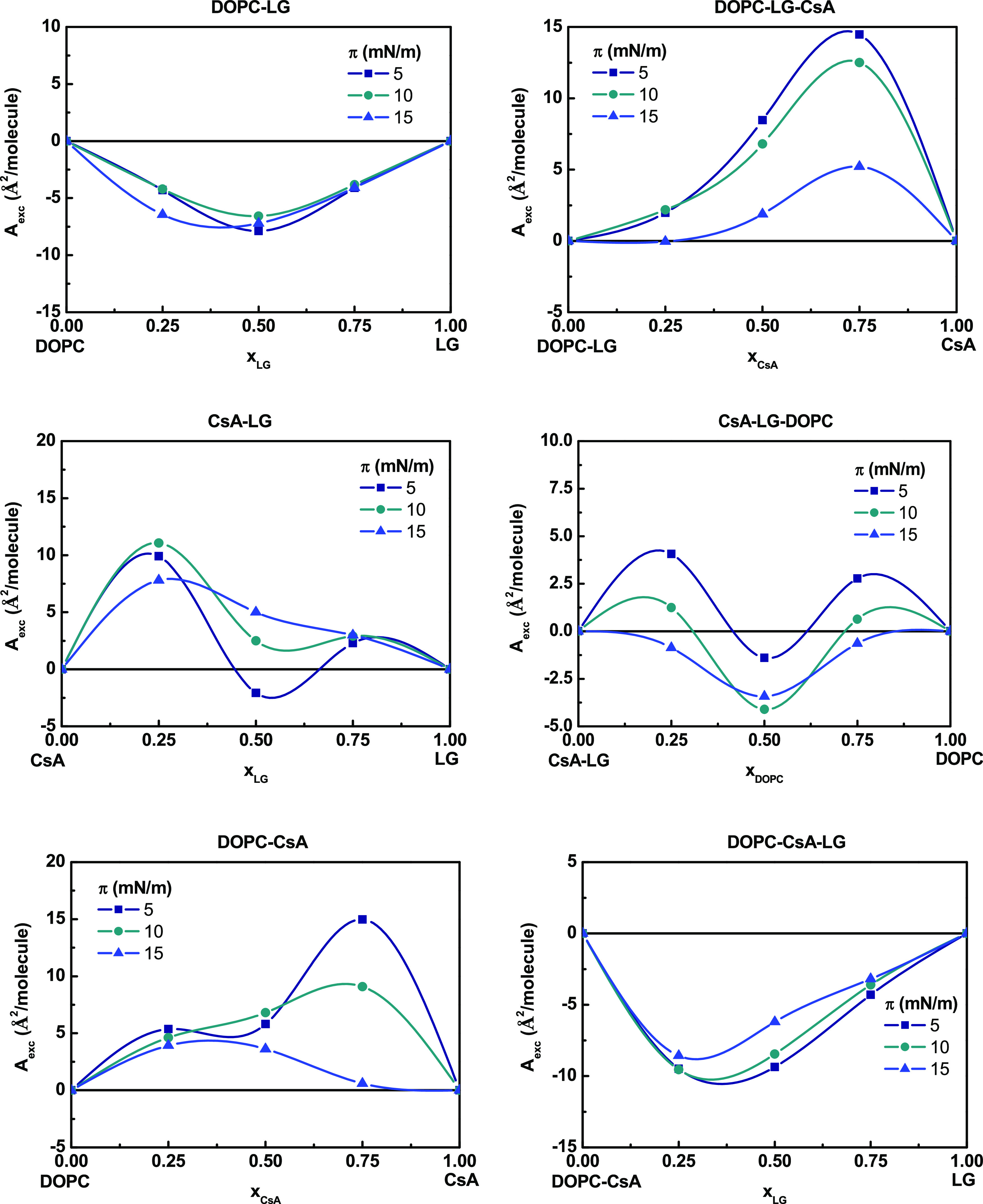
Excess area per molecule (*A*_exc_) versus composition of the binary and ternary monolayers.

**Figure 3:**
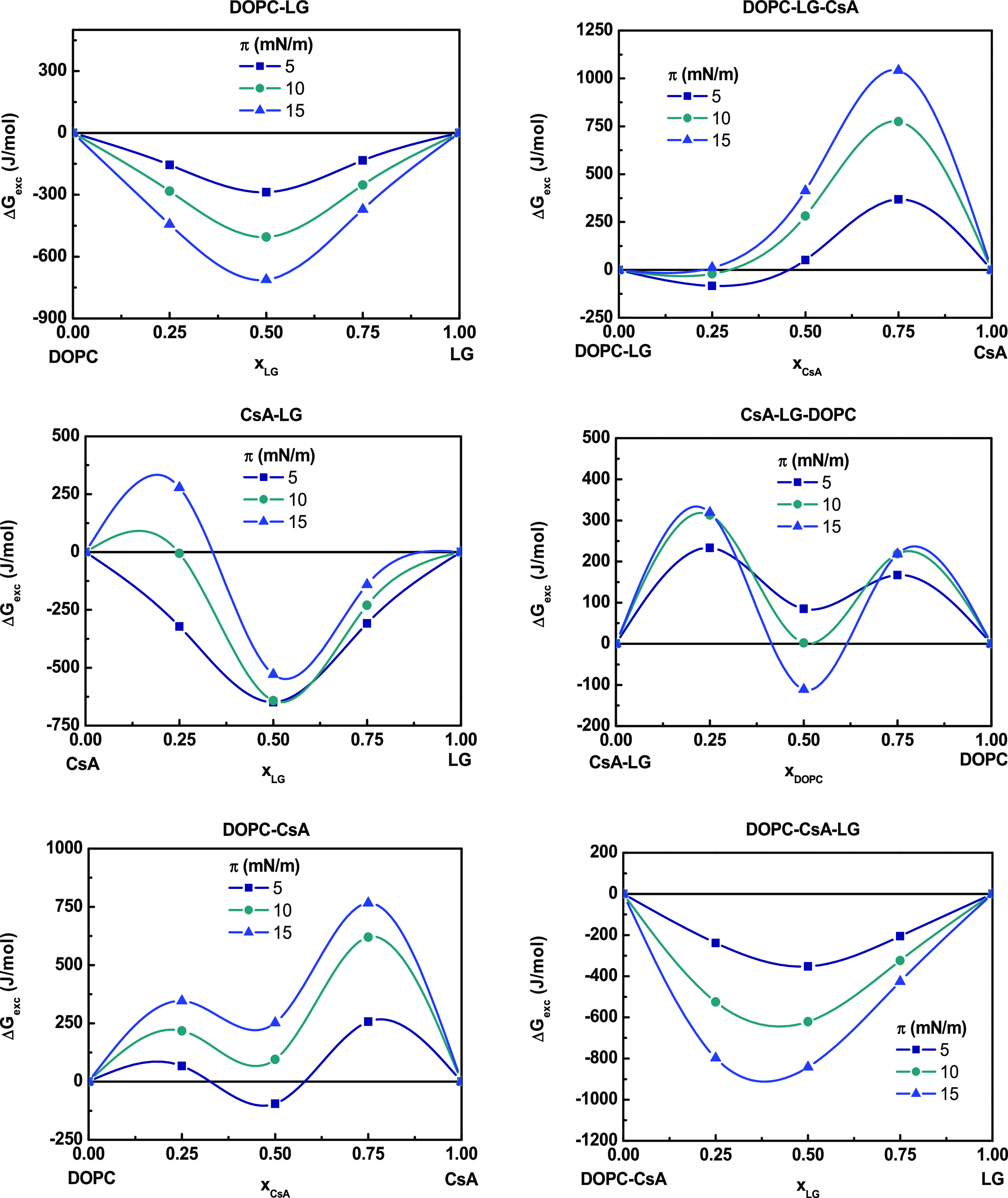
Excess Gibbs energy of mixing (Δ*G*_exc_) versus composition of the binary and ternary monolayers.

**Figure 4:**
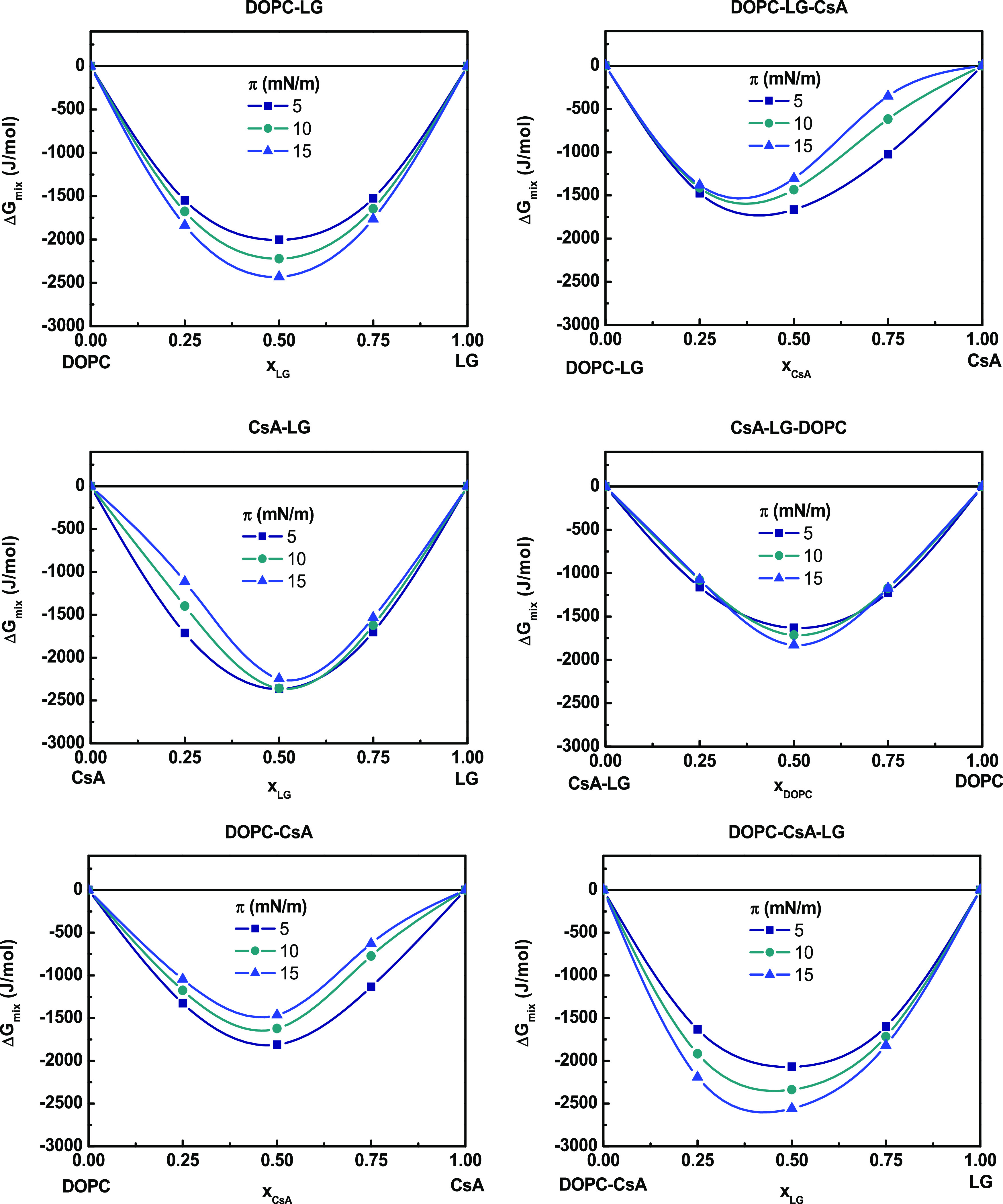
Total Gibbs energy of mixing (Δ*G*_mix_) versus composition of the binary and ternary monolayers.

The miscibility analysis for multicomponent monolayers was conducted at the surface pressure of 5, 10, and 15 mN/m. These selected surface pressures are lower than the collapse surface pressure for a CsA monolayer; therefore, the presence of all three components in the monolayer is ensured. The results are presented in Figures 2–4. Negative values of the excess area and Gibbs energy of mixing indicate the presence of attractive interactions between the molecules, which stabilize the mixed monolayer. Conversely, positive values point out the repulsive interactions that destabilize the system (demixing). The magnitude of these interactions increases with the absolute value of the Gibbs energy.

#### Binary Monolayers

The thermodynamic analysis of the interactions in the qualitative and quantitative aspects proves that for binary mixtures, the attractive interactions in the 5–15 mN/m range of surface pressures occur only between DOPC–LG as indicated by the negative values of *A*_exc_ and Δ*G*_exc_ (Figures 2 and 3). The negative *A*_exc_ values point out the formation of a more compact monolayer than the ideal one. The strongest attraction forces between DOPC and LG occur for the monolayer prepared from a molar ratio of 1:1 (Δ*G*_exc_ = −712.8 J/mol, *x*_LG_ = 0.50 at π = 15 mN/m), suggesting that there is a particularly favored organization of components at this composition, which is in very good agreement with the previous results.^34^ For this mixture, as it has been observed previously, the lauryl chain of LG is located in parallel to the oleoyl chains of the DOPC, with the hydroxyl group residing vicinal to the DOPC ester carbonyl groups.^41^

In the case of CsA–LG monolayers, a change in the nature of interactions can be noticed depending on both the ratio of the compounds and the surface pressure of the monolayer. At *x*_LG_ = 0.25, positive values of *A*_exc_ and Δ*G*_exc_ are indicative of repulsive forces between the two components, which can lead to phase separation or partial miscibility. At larger molar fractions of LG, the negative Δ*G*_exc_ values suggest attraction forces, albeit the strength decreases with pressure. For CsA–LG monolayers with *x* = 0.50, the interactions are only slightly less attractive (Δ*G*_exc_ = −529.2 J/mol,) as compared to DOPC–LG, while at lower LG content (*x*_LG_ = 0.25) and at high surface pressures, the molecules repel each other (Figure 3). CsA occupies a much larger area than LG and forms a more rigid structure (higher *C*_s_^–1^, Table 2). The addition of LG to CsA makes the rings intercalated, which fluidizes the polypeptide monolayer and ensures the surface pressure- and composition-dependent miscibility. Repulsive interactions dominate in DOPC–CsA monolayers, especially at high CsA molar fractions, as indicated by the positive *A*_exc_ values (Figure 2). The magnitude of these repulsive interactions increases with increasing surface pressure (Δ*G*_exc_ = 765.6 J/mol, *x*_CsA_ = 0.75, at π = 15 mN/m), Figure 3. The presence of two maxima separated by a minimum in both *A*_exc_ and Δ*G*_exc_ versus *x*_CsA_ graphs may be indicative of the formation of domains rich in one or the other component, which reveals partial miscibility of the components.^42,43^

In addition, the total Gibbs mixing energy, Δ*G*_mix_, takes negative values (min Δ*G*_mix_ ∼ −(1.5–2.5) kJ/mol) for all binary monolayers, which confirms their thermodynamic stability, with the less negative values observed for the DOPC–CsA monolayer (Figure 4). Since all binary mixtures show the strongest attractive or the weakest repulsive interactions at a 1:1 molar ratio (*x* = 0.50) (Figure 3), the mixed three-component monolayers were analyzed in relation to equimolar two-component systems. The effect of different amounts of the third component on changes of these interactions and the overall stability of the mixed monolayers were tested.

#### Ternary Monolayers

By keeping a constant DOPC–LG 1:1 ratio and by modifying the proportion of CsA, the attractive interactions are reduced at *x*_CsA_ = 0.25; meanwhile, at higher ratios, these interactions are more repulsive and more intense as surface pressure increases (Figure 3). The *A*_exc_ positive values (Figure 2) reveal an intermolecular spacing larger than in the ideal monolayers, that is, more expanded films as reflected by the lower *C*_s_^–1^ values obtained (Table 2).

Considering the DOPC–LG–CsA 0.50 mixture, there are 2 mol of CsA per mol of DOPC and per mol of LG. Therefore, CsA competes with DOPC to interact with LG. Thus, DOPC that is not involved in the interactions with LG (free) interacts with the excess of CsA, and particularly at the CsA–DOPC ratio 3:1 the most repulsive interactions take place. Hence, the overall stability of the ternary monolayer, expressed by Δ*G*_mix_, is smaller (Δ*G*_mix_ = −1.3 kJ/mol at π = 15 mN/m) than for the binary mixture (Δ*G*_mix_ = −2.4 kJ/mol at π = 15 mN/m) and decreases with the pressure as the repulsive forces increase (Figure 4). However, the ternary systems are still thermodynamically stable, although less favorable interactions may lead to partial miscibility. In consequence, domains integrated by a certain ratio of the components appear, with some of these domains being enriched in CsA, which explains the first collapse observed in the mixed monolayers.

The incorporation of DOPC into the CsA–LG (1:1) mixtures results in an increase of the Δ*G*_exc_ values, revealing the appearance of repulsive interactions (Figure 3). In addition, the *A*_exc_ and Δ*G*_exc_ vs composition plots reveal two maxima at low and high DOPC amounts, suggesting that the ternary films are not fully miscible and phase separation can occur. If CsA–LG–DOPC 0.50 mixture is considered, there are 2 mol of DOPC per mol of CsA and per mol of LG. Therefore, the competence between DOPC and CsA to interact with LG can show that some of the CsA molecules are free to interact with DOPC, contributing to repulsion (DOPC–CsA 3:1). The overall stability of the ternary monolayers expressed by the Δ*G*_mix_ values (−1.8 kJ/mol at 15 mN/m) is lower as compared to the CsA–LG monolayer (Figure 4).

The addition of LG to the DOPC–CsA system results in a change in the sign of *A*_exc_ and Δ*G*_exc_ that moves from positive to negative values (Figures 2 and 3). This result indicates larger miscibility of the components due to the appearance of attractive interactions between the molecules, which increases with increasing the surface pressure, reaching the highest value (Δ*G*_exc_ = −841.7 J/mol) for *x*_LG_ = 0.50 at π = 15 mN/m. In addition, the determined values of Δ*G*_mix_ in the ternary mixture indicate an increase in the stability of three-component systems upon the addition of LG to the DOPC–CsA system, with more negative Δ*G*_mix_ values with increasing surface pressure (Δ*G*_mix_ = −2.6 kJ/mol at π = 15 mN/m). In contrast, for the DOPC–CsA monolayer, the tendency is the opposite one, i.e., less Δ*G*_mix_ negative values and thus less stability, with the increasing surface pressure (Δ*G*_mix_ = −1.5 kJ/mol at π = 15 mN/m).

In the DOPC–CsA–LG mixtures, both DOPC and CsA, due to dominance of the repulsive interactions, can be in a free form, which certainly facilitates interactions with LG. Since LG has an affinity for both compounds, it can be expected to interact competitively with both. Moreover, due to repulsion between DOPC–CsA, the monolayer can contain loose spaces that can be filled with LG. Thus, LG becomes a linker between DOPC and CsA molecules. As a result of the increase in molecular packing during compression, CsA adopts a more vertical orientation, which favors interactions between molecules through hydrogen bonding and Lifshitz-van der Waals forces. The LG heads have been found to locate near the DOPC ester bonds to be closer to the unsaturated bonds.^41^ This is due to the role of LG as an antioxidant. In brief, the interactions of CsA with DOPC depend on the presence of LG and change with the LG content.

### Surface Potential–Area (Δ*V*–*A*) Isotherms

**Figure 5:**
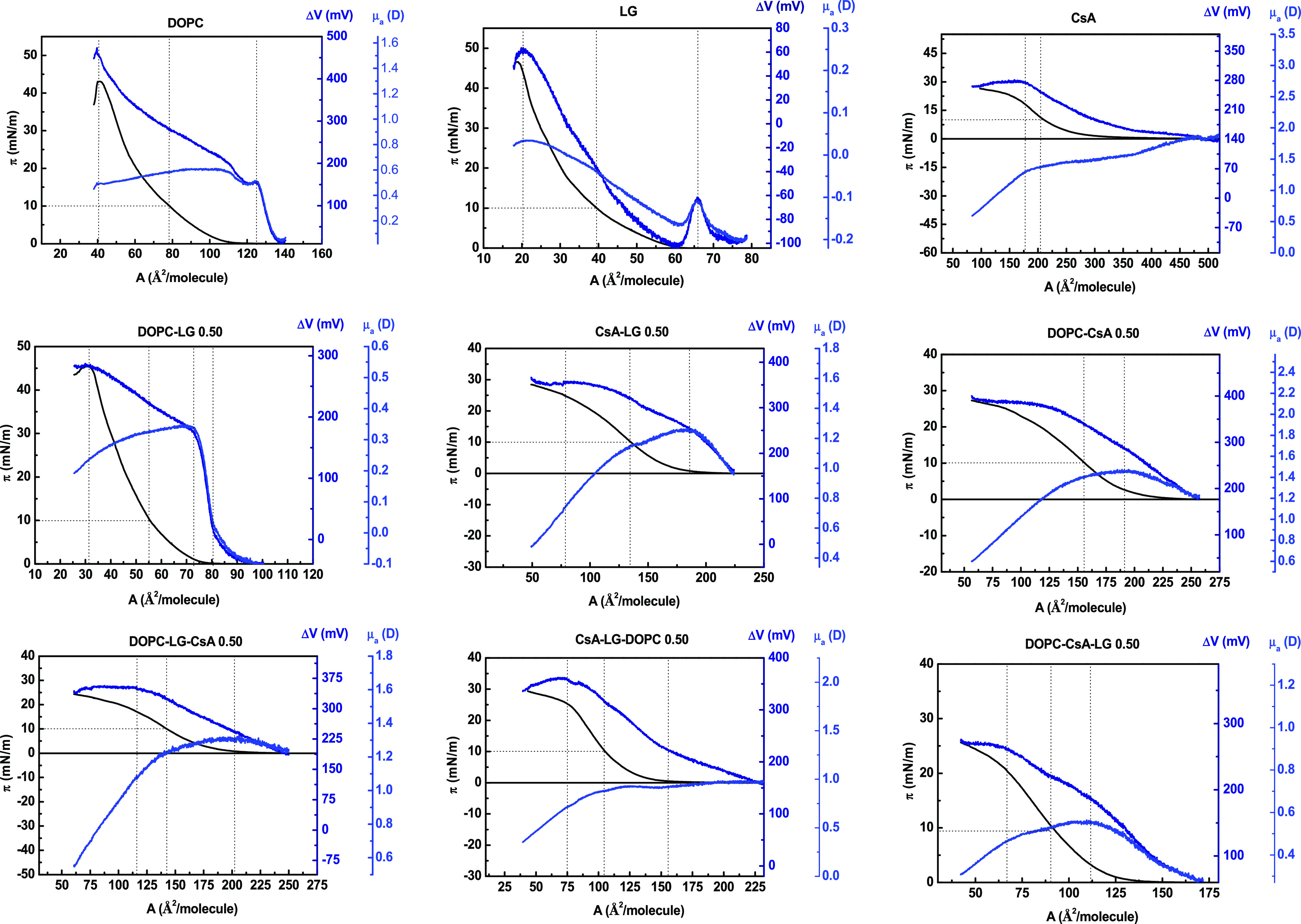
Surface pressure, π-, electric surface potential, Δ*V*-, and apparent dipole moment μ_a_-, area, *A*, isotherms of the representative monolayers.

**Figure 6:**
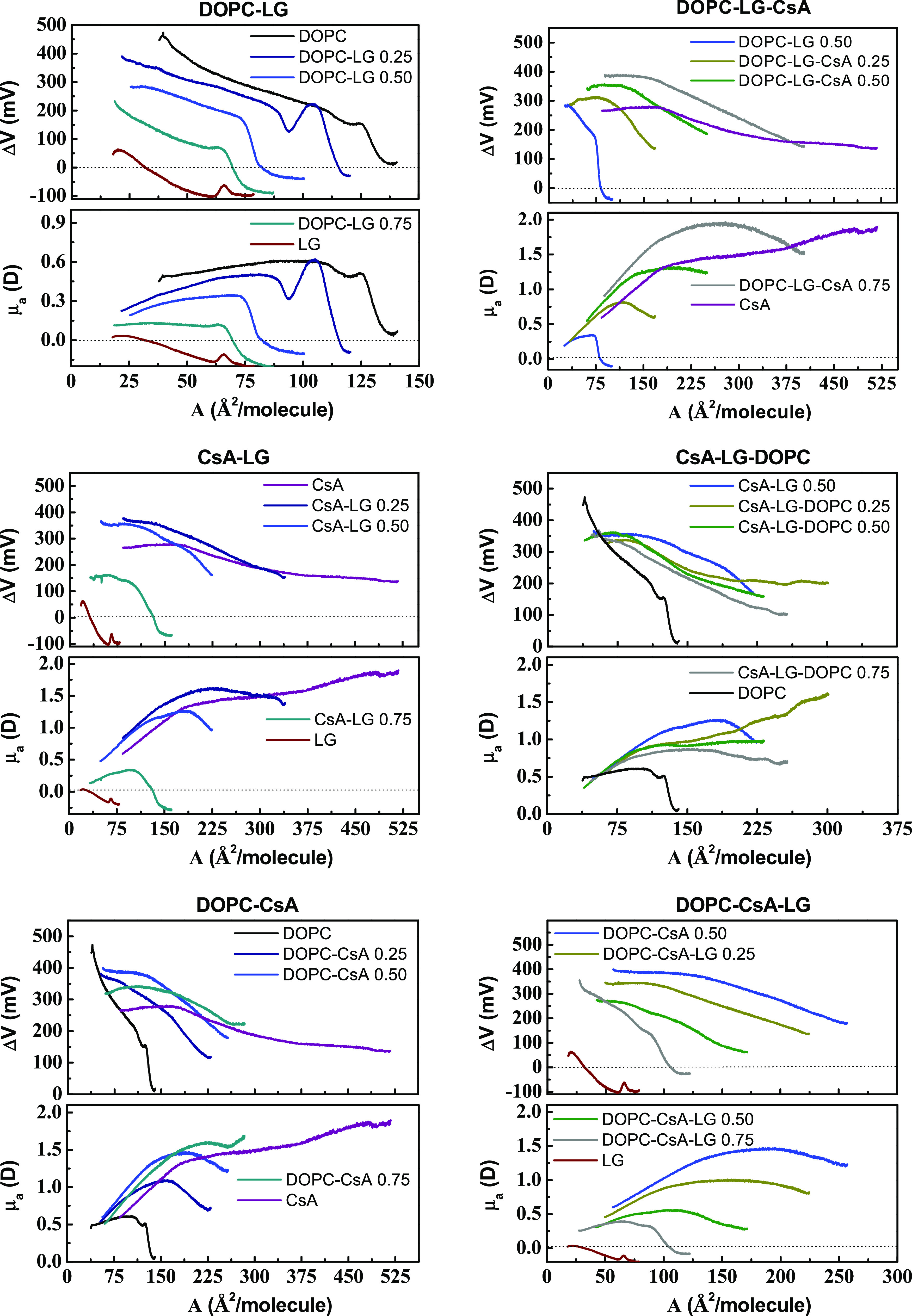
Surface potential–area per molecule (Δ*V*–*A*) and apparent dipole moment-area per molecule (μ_a_–*A*) isotherms for the single, binary, and ternary monolayers for the indicated molar fractions of components.

The surface potential Δ*V* of a monolayer is termed as the difference in potential between a clean water surface and a monolayer-covered surface.^44,45^ This quantity depends on both the packing density and the orientation of the molecules. The monolayer at the air–water interface can be treated as a set of molecular dipoles, which contributes to polarization within the film. To correlate the measured surface potentials with the dipole moments of the molecules in the monolayer, the Helmholtz equation can be used (eq 7)^36,45,46^
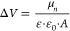
7where μ_*n*_ denotes vertical component of the dipole moment, i.e., effective dipole moment, *A* is the subphase area per molecule in the monolayer, ε is the monolayer permittivity, and ε_0_ is the vacuum permittivity (8.85417 × 10^–12^ F/m). Since ε is unknown, the changes of the effective dipole moment for the monolayer-forming molecules during compression can be expressed as the so-called apparent dipole moment, eq 8
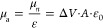
8Figure 5 shows the surface pressure, π, electric surface potential, Δ*V*, and apparent dipole moment, μ_a_, versus area, *A*, isotherms of some representative monolayers. For the remaining monolayers, these isotherms are presented as supplementary data (Figure S2). In addition, the set of surface potential changes and apparent dipole moment versus area (Δ*V*–*A* and μ_a_–*A*) for particular binary and ternary systems is presented in Figure 6.

For all monolayers, solely a fluid phase can be identified whose surface potential depends sensitively on the monolayer components and their molar fractions in mixtures (Figures 5 and 6). As the monolayer is compressed, the surface potential alters due to the pressure-induced orientation changes of the polar (heads) or nonpolar (tails) groups. The surface potential begins to increase at the area associated with the formation of a hydrogen bond network between water and polar groups of film molecules.^45^

For all fluid monolayers, the slope of the Δ*V* curve increases more or less linearly with decreasing molecular area proving changes in the orientation of molecules. Some inflections occur at areas close to *A*_0_, revealing the transition from gas to liquid-expanded (G-LE) phase driven by changes in the monolayer density when the orientation of the hydrocarbon chains becomes more perpendicular to the surface. Conversion of the Δ*V*–*A* data into μ_a_–*A* plots (Figures 5 and 6) points out that the dipole moment reaches the maximum values for G-LE phase transition and then decreases on compression when the monolayers are in the liquid-expanded phase. This decrease is even sharper when the mixed films contain CsA. Only in the case of LG, μ_a_ of molecules in the fluid monolayer gradually rises up to its collapse.

As can be clearly seen in Figures 5 and 6, the apparent dipole moment values depend on the monolayer composition. For DOPC–CsA monolayers, μ_a_ increases as the amount of CsA increases, while for DOPC–LG and CsA–LG the decrease in μ_a_ values is observed with increasing the LG molar fractions. Similar relations are obtained for the ternary monolayers. When LG is added to DOPC–CsA, μ_a_ decreases, whereas the introduction of CsA to DOPC–LG contributes to the rise of μ_a_. That is, the μ_a_ values are imposed by the potential of the added component. DOPC and CsA exhibit positive surface potential values, while LG has negative surface potential values at high areas per molecule and positive values at low areas per molecule. As mentioned above, the μ_a_–*A* isotherms possess maxima attributed to the G-LE phase transition (Figures 5 and 6) and whose position depends on the monolayer composition. Consistent with the π–*A* isotherms, the maxima shift to larger or smaller areas as the molar fractions of CsA or LG increases, respectively, in the binary or ternary systems.

As DOPC, LG, and CsA have a net dipole moment, the monolayer can be treated as a system of dipoles, each containing a component perpendicular to the air–water interface, which contributes to electrostatic forces of long range. For a DOPC monolayer, the major contribution to the surface dipole moment is due to the polarization near the hydrophobic region of the membrane, where the carbonyl dipoles are mainly responsible for determining the surface potential.^47,48^ The G-LE transition is revealed by the inflection in the Δ*V*–*A* and μ_a_–*A* isotherms (Figures 5 and 6). Then, the surface potential increases continuously with pressure due to the increase in molecular density, while the apparent dipole moment decreases as a result of a possible rearrangement in the region of head groups.^47^ In the case of a LG monolayer, the phase transition to a fluid phase is accompanied by abrupt potential and dipole moment change, followed by a gradual increase in the density of the fluid phase up to the monolayer collapse. This transition can be ascribed to a reorientation of the aromatic moiety with −*OH* groups to a more vertical position with respect to the air–water surface.

As discussed above, while DOPC has a positive surface potential, LG exhibits negative surface potential values at high areas per molecule and positive values at low areas per molecule. Different signs in the surface potential are indicative of the opposite direction of forces in electric fields.^48^ The polarization of the carbonyl groups in the DOPC confers the positive contributions of neutral phosphocholine (PC) head region to the surface potential. Meanwhile, the other PC head groups are of minor relevance since they are immersed into the water; therefore, they are strongly screened owing to the high dielectric constant and conductivity of the subphase.^48^ Thus, the >C=O groups residing in the hydrophobic chain region are the potential determining groups, responsible for the strong dependence of dipole moment on surface pressure. More precisely, the >C=O dipole changes its tilt with respect to the air–water interface upon the compression process, which has an impact on the adjacent groups by compensating the dipole moments of the interacting molecules. In consequence, depolarization of the film surface takes place. Moreover, both DOPC and LG are amphiphilic compounds containing two unsaturated C18 chains or a saturated C12 hydrocarbon chain, respectively. Upon compression, these hydrocarbon chains are more vertically oriented with respect to the surface, which contributes to the surface potential but simultaneously affects the carbonyl group orientation changes. As for CsA, it contains many peptide bonds, thus bearing a net of dipoles. The intrinsic charge distributions, where nitrogen has a partial positive charge and the oxygen has a partial negative charge, generate the increased value of the apparent dipole moment and H-bond formation when CsA lies on the water surface. Upon compression, the molecules change their orientation and/or conformation so that the dipole moments are successively compensated. In consequence, the more or less linear decrease in the dipole moment with pressure is due to a reorientation of the potential determining groups within the film.^45^ Another reason for this decrease can be found in the surrounding water dipoles, which can screen forces coming from the head groups.^48^ The same interpretation can be applied for the mixtures where, additionally, the interactions between molecules also can affect the apparent dipole moment changes.

### Molecular Interactions between Components

Taking together the findings presented in this paper as well as those published previously the probable mechanism of molecular interactions between DOPC, CsA, and LG is discussed below.

To penetrate the membrane, CsA molecules have to be conformationally flexible to reduce the entropic cost of their insertion.^49^ CsA is the 11-residue cyclic peptide, which does not have ionizable functional groups and is slightly soluble in water. Its lipophilic nature is achieved through extensive methylation of the amino acid residues (seven N-methylated moieties).^6,9^

The conformational changes in a drug depend on the dielectric constant of the solvent.^50^ For instance, the dominant conformation in chloroform is conditioned by the presence of intramolecular H-bonds stabilizing the secondary structure. In polar solvents, the molecule exposes its H-bonding groups and loses its secondary structure becoming a kind of single-molecular micelle with a higher affinity to water molecules than it would be expected.^9^

These facts constitute the basis of the below discussion on the behavior of CsA at the air/water interface and its adaptation in the DOPC and/or LG monolayers, including interactions between the components.

Specifically, the conformational flexibility of CsA denotes that its conformation changes dynamically when transitioning between high and low dielectric environments.^50−52^ These conformational transitions are dealing with intramolecular hydrogen bonding. In water and at water/membrane interface, CsA exists in an optimal “open” conformation with the lowest solvation energy. Then, the CsA molecule is round-shaped and can form only one intramolecular hydrogen bond^52^ or does not form any.^51^ The backbone amides are free to interact with water via hydrogen bonds. In this type of interaction, the −OH group of the most flexible part of CsA, i.e., the MeBmt-1 amino acid, is also engaged.^29^ On the other hand, in a lower dielectric hydrophobic environment, such as hydrocarbon chains, CsA is assumed to have a conformation of the crystal structure.^53,54^ Moreover, the MeBmt-1 amino acid is folded over the rest of the CsA molecule to be positioned deep into the membrane.^29^ Therefore, the energetically preferred conformation in the middle of the membrane is a closed one of elongated oval-shaped stabilized by three or four intramolecular H-bonds.^50−52^ Hence, CsA transitions into a “closed” conformation result in the reduction of the CsA effective polar surface area.^49^

It is likely that before compression, the cyclic amino acid ring of CsA is positioned in the water surface plane with hydrophobic side chains sticking out to the air. Such a state corresponds to the “open” conformation in which the −OH, >C=O, and >N–H groups participate in hydrogen bonding with both the water and head groups of other molecules. Under compression, the rings approach each other to form a rigid monolayer^55^ as revealed by the increased compression modulus (Table 2). The monolayer stiffness is due to strong *cis*-amide and hydrogen bonding within the amino acid ring, while the side chains are still flexible.^54^ At the same time, the rings can change their orientation from parallel to a more vertical orientation with respect to the surface.^35^ This behavior is expressed in the surface potential increase with a simultaneous decrease of the apparent dipole moment. Interestingly, this decrease is even sharper when the surface potential *plateau* appears in the region of collapse.

When CsA is accompanied by DOPC and/or LG molecules and the monolayer is compressed, intermolecular interactions start to appear through hydrogen bonding between the backbone amides and/or hydroxyl group of CsA and H acceptors from the lipid oxygen atoms and/or H-donors from the phenol head groups. In such a case the open structure of CsA is preferred. Thus, the PC and PG groups can be perturbed by the CsA molecules at the air–water interface.^29^ However, the situation is completely different when during compression the CsA molecule undergoes a change in orientation. The hydrocarbon chains of DOPC and/or LG determine a hydrophobic environment (which is equivalent to a low dielectric constant medium) for CsA, which favors its transition from an open to a closed conformation. Thus, the polar groups of the polypeptide are engaged in the intramolecular H-bonds, while the hydrophobic residues interact with the region of tails by hydrophobic interactions.

The main characteristics in the Δ*V*–*A* and μ_a_–*A* isotherms are inflections at areas per molecule, corresponding to the onset of a G-LE phase transition, which indicates a nearly horizontal slope of the π-A isotherms (Figures 5 and 6). A specific inflection on the Δ*V*–*A* isotherms corresponds to the maximal apparent dipole moment. The ascending branch of the plot, where an increase in the dipole moment is observed, can be attributed to the process of taking the more upright position by molecules. This effect is probably intensified mutual polarization of the dipoles as the film becomes more tightly packed as well as due to the structural rearrangement within the head group region. Further compression causes a gradual depolarization of the monolayer as depicted by the falling branch.

**Scheme 2:**
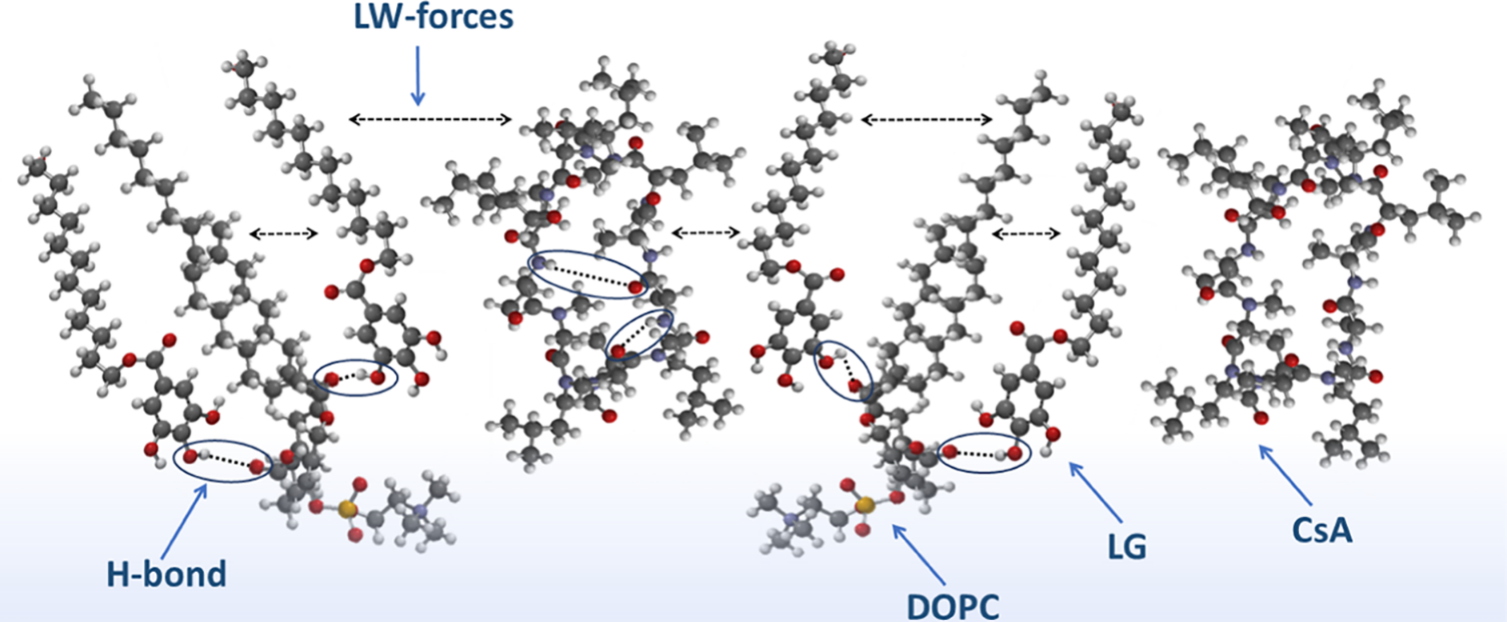
Possible Alignment of Molecules in the Most Packaged State of DOPC–CsA–LG 1:1:2 Monolayer, where DOPC, 1,2-Dioleoyl-*sn*-glycero-3-phosphocholine; CsA, Cyclosporine A; LG, Lauryl Gallate; H-Bond, the Hydrogen Bonding; and LW Forces, the Lifshitz-van der Waals Forces

Interestingly, when the molar ratio of the DOPC–CsA–LG mixture is 1:1:2, the increased content of LG may result in an alignment of molecules in the monolayer in which DOPC and CsA are separated by LG molecules as shown in Scheme 2. This schematic model provides a visual depiction of the possible distribution of molecules in the most packaged state of a DOPC–CsA–LG 1:1:2 monolayer, below the first collapse. The optimal conformation of each individual molecule was simulated using Spartan 08 V 1.2.0. It should be emphasized that the particular conformation corresponds to the free molecule, not to the mixture, but they are used jointly to visualize the mixed monolayer.

The model illustrates intra- and intermolecular H-bonds as well as Lifshitz-van der Waals (LW) forces between components, which play an essential role in the monolayer properties. In the DOPC molecule, the oxygen sites available as H acceptors are located at the phosphate (−OPO_3_^–^−) and the two carbonyl (>C=O) groups. However, H-donor hydroxyl groups of LG, favoring interactions with the carbonyl groups of DOPC,^41^ presumably show that the LG head groups point toward the hydrophobic region of the monolayer, thereby lauryl tails reside in the fatty acid chain zone. In the hydrocarbon chain environment, CsA preferentially exists in the closed conformation with intramolecular H-bonding, interacting with DOPC and LG mostly by LW forces.

It should be stressed that due to lipophilicity, CsA can localize in the interior of the DOPC membrane perturbing the acyl chain region, which is close to the head groups.^28,29^ The same region of DOPC can be occupied by LG. Therefore, the competition for the same binding sites occurs and simultaneously the fatty acyl chain flexibility is limited as a result of the restrictions of their conformational freedom.^29,56^ Such a process promotes the formation of stable monolayers. Due to steric hindrance, when the conformational freedom is restricted, the structure of such composition is mostly favored facilitating the mutual interactions as it provides the least Gibbs free energy, that is, highest stability (Figures 3 and 4). At other proportions, such strong attractive interactions are not obtained, presumably due to the fact that the suitable structural arrangement is not ensured. The lack of optimal spatial matching of the interacting molecules can impede the specific interactions between them and even lead to phase separation. This scenario is plausible for the mixed monolayers exhibiting positive values of the excess Gibbs energy of mixing (Figure 3).

Owing to the affinity of LG for DOPC (negative *A*_exc_ and Δ*G*_exc_, Figures 2 and 3, respectively), its inclusion in the film causes a reduction of the free volume inside the hydrophobic part of DOPC producing an increased packing density as expressed by *C*_s_^–1^ for DOPC–LG (Table 2). Hence, the access of CsA to carbonyl group sites of DOPC is impeded and probably the CsA molecules are arranged in parallel along the long axis of the LG and/or DOPC molecules.^57^ The specific DOPC–CsA interactions can be manifested as the pressure-induced changes in the orientation and/or conformation which, in addition, are sensitive to the LG content, thus contributing to the membrane stability.

The mechanism of the DOPC–CsA monolayer stabilization by LG can also be connected with its antioxidant activity. LG associates preferentially with unsaturated phospholipids and resides closer to the double bonds, i.e., LG is deeply submerged within the DOPC monolayer toward air so that its pyrogallol (PG) group is situated near the ester groups of DOPC, while the lauryl tail is located among the oleoyl chains (Scheme 2). Such a possible colocalization of molecular moieties in the binary DOPC–LG monolayers was confirmed previously.^41^ LG strengthens the stability of the mixed monolayers by interacting the hydroxyl groups of PG with the polar parts of PC, particularly with >C=O, through hydrogen bonds. Then, the carbonyl groups of DOPC, and in general the acyl chain regions, are less affected by CsA, whose ability to adopt a closed conformation increases. By shielding the polar groups from an apolar environment, typically through intramolecular hydrogen bonds, CsA is able to penetrate the hydrophobic core and interact with the hydrocarbon chains present in DOPC and LG mainly by the Lifshitz-van der Waals forces (Scheme 2). From a biological point of view, this process seems to be beneficial for drug–membrane permeability.^49^ LG does not rather penetrate the hydrocarbon matrix but being at the lipid/water interface, near unsaturated bonds of DOPC, provides the protective barrier against oxidation due to the reduction of the oxidants’ access to the film.

## Conclusions

In this contribution, surface pressure versus area per molecule isotherms (π–*A* isotherms) for one- and multicomponent Langmuir monolayers incorporating the phospholipid DOPC, the immunosuppressant CsA, and the antioxidant LG were recorded at the air–water interface. The physical state (packing and ordering), miscibility, and thermodynamic stability of binary and ternary monolayers of varying composition were evaluated from these isotherms and thermodynamic parameters (excess area, excess Gibbs energy, and total Gibbs energy of mixing) were determined. In addition, information about orientation changes of molecules upon the compression process was obtained from surface potential measurements and apparent dipole moment changes.

The data consistently show that all three components interact below the first collapse pressure, albeit the mixtures differ in the strength of mutual interactions depending on the molecular organization and composition. It should be emphasized that the ratio of the components in the monolayer has a high impact on their interactions (miscibility) promoting beneficial arrangement in the fluid phase. Differences in mixing properties were illustrated by a comparison of the Δ*G*_exc_ and Δ*G*_mix_ values versus composition and surface pressure. The possible steric perturbations in the middle of the acyl chain region of DOPC due to the cyclic CsA ring do not result in total demixing but partial miscibility, as confirmed by the negative values of Δ*G*_mix_. The addition of LG molecules to the DOPC–CsA monolayer induces more attractive interactions in the ternary systems, thereby they become thermodynamically more stable.

Understanding the molecular interactions between DOPC–CsA–LG is mandatory for a correct choice of composition for implant coating. According to the results presented here, three mixtures, DOPC–CsA–LG 0.25, 0.50, 0.75, would be suitable for the preparation of a stable cover for the forthcoming deposition on a polymer-based implant surface. In the next step, we will focus our investigations on the detailed characterization of solid-supported multicomponent layers using a set of different physicochemical methods.

## Supporting Information Available

Compression modulus (*C*_s_^–1^) versus surface pressure (π) for the binary and ternary monolayers (Figure S1); surface pressure, π-, electric surface potential, Δ*V*-, and apparent dipole moment, μ_a_-, area, *A*, isotherms for all monolayers studied (Figure S2) (PDF)
