# Supplementary material for: Analysis of Molecular Interactions between Components in Phospholipid-Immunosuppressant-Antioxidant Mixed Langmuir Films
Source: Langmuir. 2021 Apr 29;37(18):5601–16. doi: 10.1021/acs.langmuir.1c00434 (PMC8280729; doi:10.1021/acs.langmuir.1c00434)
Supplement: Supplementary file 1 — la1c00434_si_001.pdf [file la1c00434_si_001.pdf]

Analysis of Molecular Interactions between Components in Phospholipid-Immunosuppressant-Antioxidant Mixed Langmuir Films

Małgorzata Jurak,[a*] Klaudia Szafran[a], Pilar Cea[b,c] and Santiago Martín[b,c]

[a] Department of Interfacial Phenomena, Institute of Chemical Sciences, Faculty of Chemistry, Maria Curie-Skłodowska University in Lublin, 20031 Lublin, Poland
[b] Instituto de Nanociencia y Materiales de Aragón (INMA), CSIC-Universidad de Zaragoza, 50009 Zaragoza, Spain
[c] Departamento de Química Física, Facultad de Ciencias, Universidad de Zaragoza, 50009 Zaragoza, Spain

[*]*Corresponding author (M. Jurak)*
e-mail address: malgorzata.jurak@umcs.pl
phone: +48815375547, fax: +48815375656

SUPPORTING INFORMATION

Figure S1. Compression modulus (C_S^{-1}) versus surface pressure (π) for the binary and ternary monolayers.
Figure S2. Surface pressure, $\pi -$, electric surface potential, $\Delta V -$, and apparent dipole moment, $\mu_a -$, area, A, isotherms for all monolayers studied.

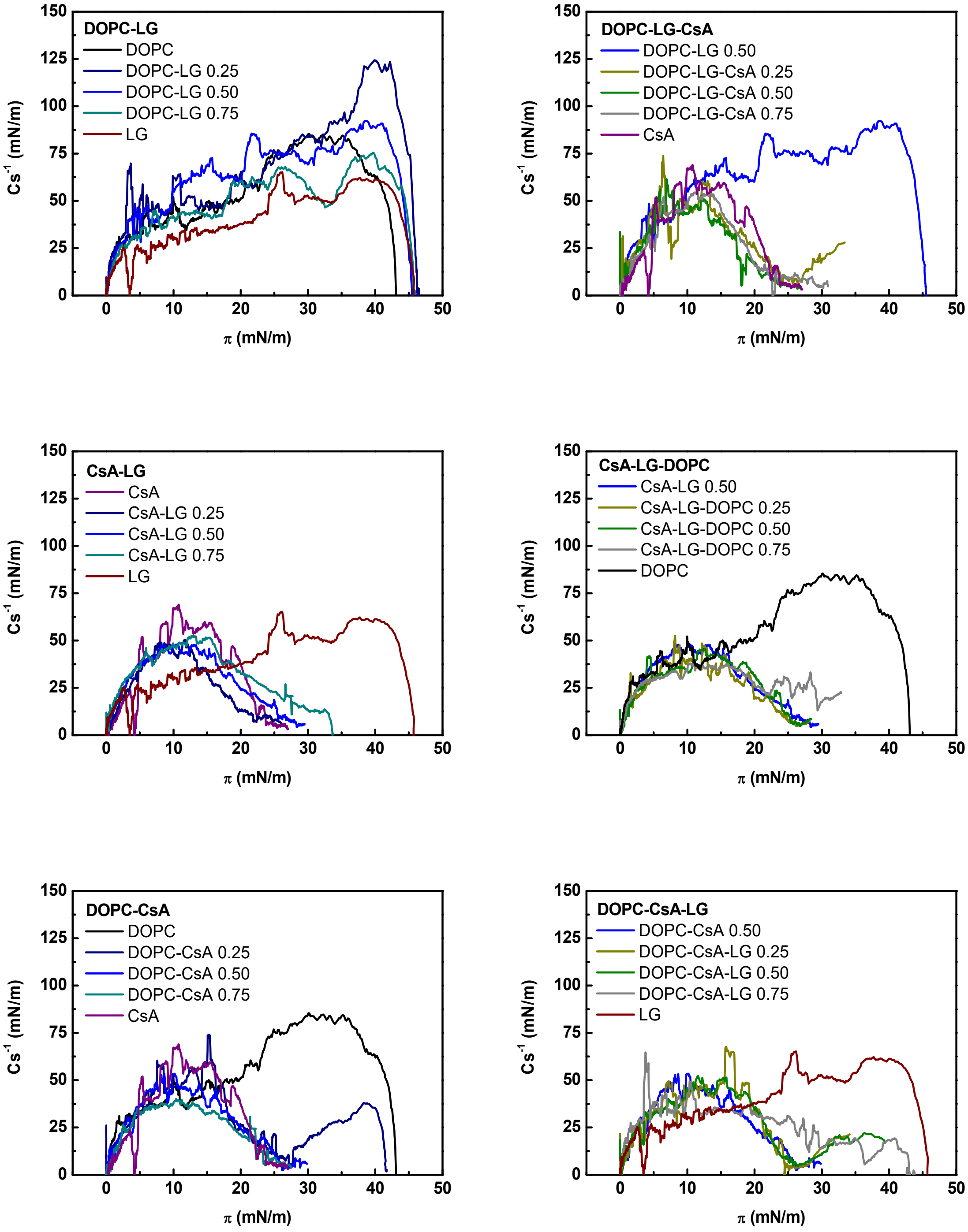

Figure S1. Compression modulus (C_S^{-1}) versus surface pressure (π) for the binary and ternary monolayers.

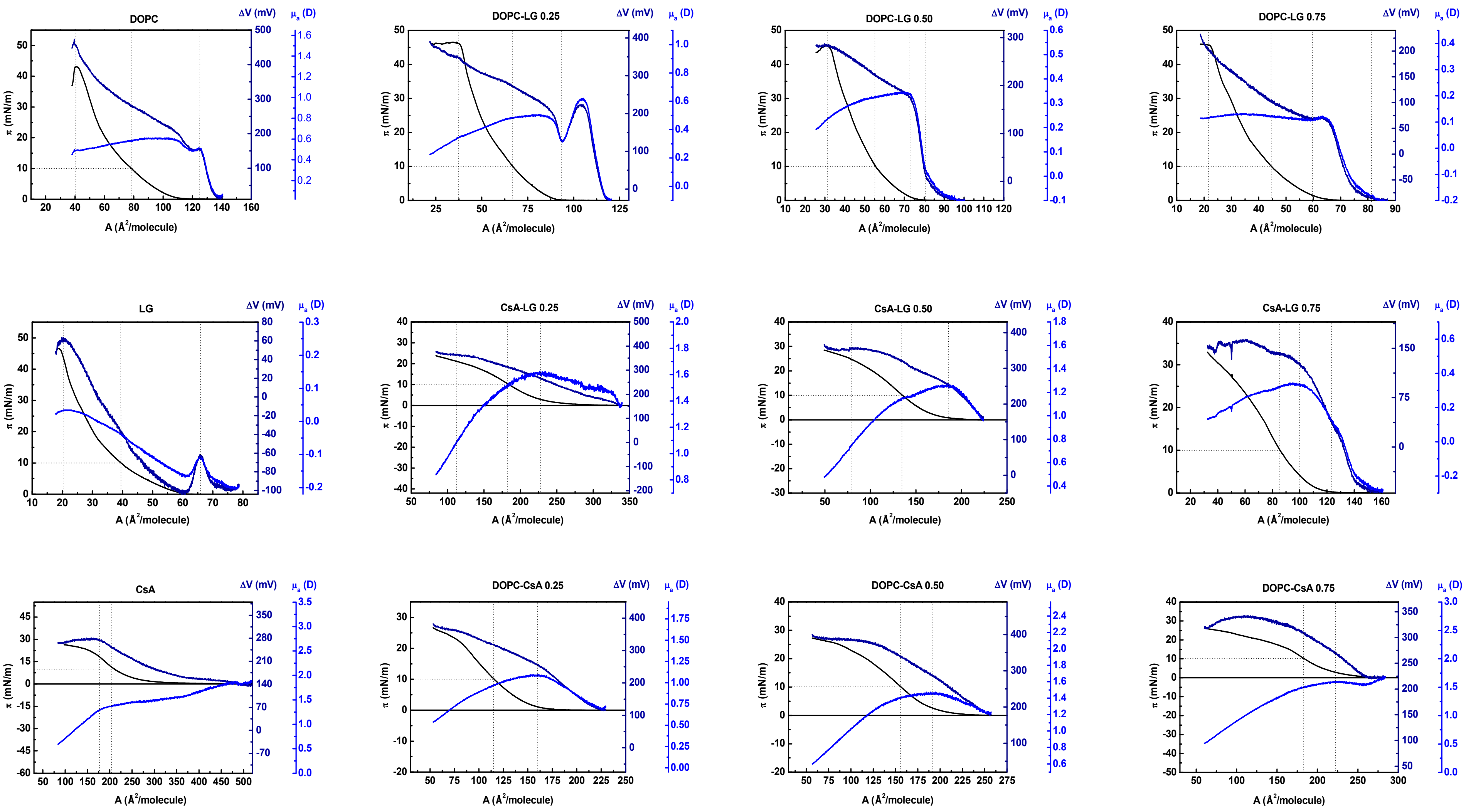

Figure S2. Surface pressure, π −, electric surface potential, ΔV −, and apparent dipole moment, μ_a −, area, A, isotherms for all monolayers studied.

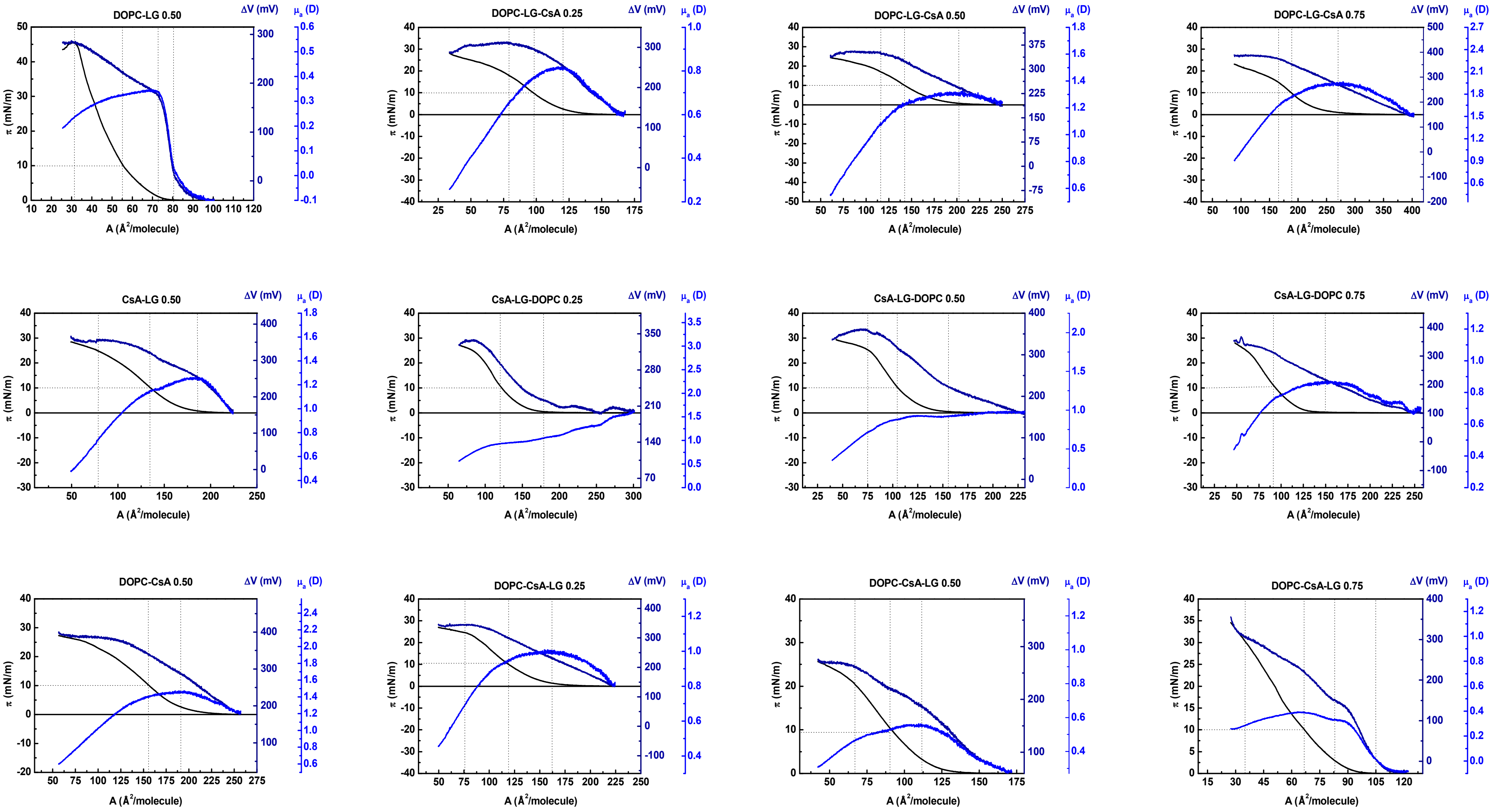

Figure S2. Surface pressure, π –, electric surface potential, ΔV –, and apparent dipole moment, μ_a –, area, A, isotherms for all monolayers studied (continued).
